# Breast cancer scoring based on a multiplexed profiling of soluble and cell-associated (immune) markers facilitates the prediction of pembrolizumab therapy

**DOI:** 10.1186/s12935-025-03729-7

**Published:** 2025-03-27

**Authors:** Verena Schweihofer, Christina Bruss, Stephan Seitz, Gunther Glehr, Madeleine Hetterich, Florian Weber, Maria Hatzipanagiotou, Miriam Fernández-Pacheco Álvarez, Olaf Ortmann, Gero Brockhoff, Richard J. Bauer, Anja Kathrin Wege

**Affiliations:** 1 Department of Gynecology and Obstetrics, University Medical Center Regensburg, Regensburg, Germany; 2 Department of Surgery, University Hospital Regensburg, Regensburg, Germany; 3 Department of Oral and Maxillofacial Surgery, University Hospital Regensburg, Regensburg, Germany; 4 Institute of Pathology, University of Regensburg, Regensburg, Germany; 5 Bavarian Cancer Research Center (BZKF), Regensburg, Germany

**Keywords:** Checkpoint expression, Checkpoint secretion, Soluble factors, Checkpoint therapy, Breast cancer (BC), Tumor phenotype, Immune checkpoint, Tumor markers

## Abstract

**Background:**

The immune checkpoint targeting is nowadays an integral part of cancer therapies. However, only a minority of patients experience long-term benefits. Thus, the identification of predictive biomarkers contributing to therapy response is urgently needed.

**Methods:**

Here, we analyzed different immune and tumor specific expression and secretion profiles in the peripheral blood and tumor samples of 50 breast cancer patients by multicolor flow cytometry and bead-based immunoassays at the time of diagnosis. Due to individual phenotype variations, we quantitatively scored 25 expressed and secreted immune-associated (e.g., LAG-3, PD-1, TIM-3, CD27) and tumor relevant markers (e.g., PD-L1, CD44, MHC-I, MHC-II) in immune checkpoint-treated triple negative breast cancer patients based on the current literature. The calculated score divided the patients into individuals with predicted pCR (total score of > 0) or predicted residual disease (total score of ≤ 0). At the end of the neoadjuvant therapy, the truly achieved pathological complete response (pCR; end of observation) was determined.

**Results:**

The calculated score was 79% in accordance with the achieved pCR at the time of surgery. Moreover, the sensitivity was 83.3%, the specificity 76.9%, the positive predictive value 62.5%, and the negative predictive value 90.9%. In addition, we identified a correlation of PD-1 and LAG-3 expression between tumor-associated and peripheral immune cells, which was independent of the subtype. Overall, PD-1 was the most frequently expressed checkpoint. However, in a number of patient-derived tumors, additional checkpoints as LAG-3 and TIM-3 were substantially (co-)expressed, which potentially compromises anti-PD-(L)1 mono-therapy.

**Conclusions:**

This study represents a proof-of-principle to identify potential checkpoint therapy responders in advance at the time of diagnosis. The work was based on a scoring derived from a multiplexed marker profiling. However, larger patient cohorts need to be prospectively evaluated for further validation.

**Supplementary Information:**

The online version contains supplementary material available at 10.1186/s12935-025-03729-7.

## Background

Breast cancer (BC) is the second most common cause of cancer-associated death in women [1], even though, the prognosis considerably varies for hormone receptor (HR^+^), human epidermal growth factor receptor 2 (HER2^+^; with or without HR expression) and HER2^-^/HR^-^ diseases, the latter classified as triple-negative breast cancer (TNBC). In early stage TNBC neoadjuvant chemotherapy (NACT) proved to be a promising strategy but meanwhile immune checkpoint inhibitor therapy (ICI) in combination with chemotherapy is a partially successful approach to treat early [2] and metastatic TNBC patients [3, 4]. In 2020, the anti-PD-1 (programmed cell death 1) antibody pembrolizumab (applied in combination with chemotherapy) was authorized for the treatment of metastatic TNBC. This approval was based on the improved progression free survival (PFS) of patients whose tumors express PD-L1 (programmed cell death ligand 1) combined positive score (CPS) ≥ 10 [5]. Based on the results of the KEYNOTE-522 trial, the use of pembrolizumab (in combination with chemotherapy) has also been approved by the Food and Drug Agency (FDA) and the European Medicines Agency (EMA) for the neoadjuvant treatment of high-risk, early-stage TNBC patients, independent of the PD-L1 status [6].

To date, PD-L1 expression on immuno-histochemistry is currently the only approved biomarker to select patients with TNBC for immunotherapy. However, its predictive value is uncertain and certainly limited as well as irrelevant in the early-stage setting. There is also the issue of intra- and inter-tumor PD-1/PD-L1 heterogeneity and the existence of various diagnostic assays. More specifically, the inhibition of the PD-1/PD-L1 axis is not always beneficial for patients with PD-L1^+^ tumors, which might be due to the co-expression of additional checkpoint molecules. Besides PD-1, the lymphocyte activation gene (LAG-3) is another transmembrane receptor, which is frequently upregulated on the surface of activated T cells. Co-expression of both receptors represents an enhanced exhausted phenotype, hence dual targeting might be an option to overcome ICI resistance [7]. The binding partner of LAG-3 is the major histocompatibility complex II (MHC-II) but also other ligands such as galectin-3 or the T cell antigen receptor (TCR)–CD3 complex. The involvement of LAG-3 mediates inhibitory activity, however, the signaling pathway is not completely understood [8].

The T cell immunoglobulin and mucin domain-3 (TIM-3) is expressed on IFN-γ-producing and intratumoral T cells, regulatory T cells (Tregs), and antigen-presenting cells (APCs) [10]. The interaction with one of its ligands, namely galectin-9 (Gal-9), has been shown to induce immune cell death, promotes tumor growth, and suppresses adaptive immune responses [11]. Other potentially relevant ligands are high mobility group protein B1 (HMGB1), carcinoembryonic antigen-related cell adhesion molecule 1 (CEACAM1), and TIM-3, which has been described as markers for exhausted and dysfunctional CD8^+^ T cell populations and natural killer (NK) cells associated to solid and hematological malignant diseases [12]. Although TIM-3 or LAG-3 expressions are associated with an exhaustion phenotype their expression has been associated with an improved outcome in different cancer subtypes including BC [11, 13, 14]. Targeting of TIM-3 to prevent or reverse exhaustion by specific antibodies is efficient in triggering anti-tumor responses and several trials evaluating mono- or combination therapies are ongoing [15]. 

Different surface molecules, including checkpoints, can undergo alternative splicing or cleavage by a disintegrin and metalloproteinase (ADAM10 or ADAM17) that causes the release of soluble variants [16]. However, the function and (clinical) importance of soluble factors are poorly understood. Nevertheless, there is some evidence for ADAM molecules to be associated with prognosis or ICI prediction. For instance, soluble CD27, which is associated with T cell activation and proliferation [17], has been defined as a negative prognostic factor in solid cancer patients undergoing ICI [18].

The identification of prognostic but more importantly predictive biomarkers is an essential step towards precision medicine and personalized treatment. Therefore, we investigated potentially highly relevant cell-associated and secreted immune profiles in BC tissue and corresponding blood samples. Finally, based on the literature, we ranked the analyzed soluble and membrane-based parameters at the time of diagnosis in TNBC patients and correlated the thereby calculated predictive score to the clinical course of the disease.

## Methods

### Patient information, treatment and sample preparation

BC biopsy and matched plasma samples from primary tumors of early stage BC patients (details are summarized in Table 1) were collected from March 2023 to July 2024 (before treatment). Single cell suspension was generated by cutting the tissue into small pieces and passing it through a 40 μm cell strainer (Falcon, Thermo Fisher Scientific, USA). Upon centrifugation (300 × g for five minutes at 4 °C) supernatant was discarded and cells were eluated in 1% FBS, 0.01% NaN3 and Dulbecco´s phosphate buffered saline (DPBS) buffer (Gibco, Thermo Fisher Scientific, USA). 100 µl of peripheral EDTA blood samples were lysed using FACS lysing solution (BD Biosciences, USA, Cat. No. 349202) and washed twice with 1% FBS, 0.01% NaN3 and DPBS buffer (300 × g for five minutes at 4 °C). The study included TNBC patients based on their nodal status (positive or negative) and/or tumor size greater than 2 cm and received neoadjuvant chemotherapy and pembrolizumab following the protocol of the KEYNOTE-522 trial [6], The pathologic complete response (pCR) or non-pCR was defined by the examination of residual tumor cells in the resected breast tissue by the pathologists (end of observation).

**Table 1:** Characteristics of 50 breast cancer tumors were analyzed in this study. *Two patients with two different subtype tumors; **two patients (three tumors) without information about TNM, G status; ***HER2: 2^+^ FISH (fluorescence in situ hybridization) negative; TNBC = triple negative breast cancer; HER2 = humane epithelial growth receptor 2 positive breast cancer; yr = year; ER = estrogen receptor; PR = progesterone receptor; Luminal B (estrogen receptors (ER^+^) and/or progesterone receptors (PR^+^)) with ki67 > 14%)

Variable	TNBC ***	HER2^+^	Luminal B	Total (BC)
	(*n* = 23)	(*n* = 6)	(*n* = 21)	(*n* = 50)*
**Mean age** – (yr)	53	53.7	50	52.1
**Sex - no. (%)**				
Male	0	0	0	0
Female	23 (100)	6 (100)	21 (100)	48 (100)*
**N stage positive (%)**	10 (45.5) **	3 (50.0)	10 (52.6)**	23 (45.1)**
**M0 / Mx (%)**	22 (100) **	6 (100)	19 (100)**	45 (100)**
**T stage (%)**				
T1	3 (13.6) **	0 (0)	3 (15.8)**	6 (13.3)**
T2	18 (81.8) **	5 (83.3)	11 (57.9)**	33 (73.3)**
T3	1 (4.5) **	1 (16.7)	3 (15.8)**	5 (11.1)**
T4	0 (0)	0 (0)	2 (10.5)**	2 (4.4)**
**Grade (%)**				
G2	13 (59,1) **	5 (83.3)	12 (63.2) **	29 (64.4)**
G3	9 (40.9) **	1 (16.7)	7 (36.8) **	17 (37.8)**
**Clinico-pathological markers**				
ER positive > 2 (%)	0 (0)	0 (0)	21 (100)	21 (43.8)
PR positive > 1 (%)	0 (0)	0 (0)	20 (95.2)	20 (41.7)
HER2neu Dako: 0 (%)	16 (69.6)	0 (0)	5 (23.1)	21 (43.8)
HER2neu Dako: 1+ (%)	6 (26.1)	0 (0)	6 (28.6)	12 (25.0)
HER2neu Dako: 2+/3+ (%)	1 (4.3) ***	6 (100)	10 (43.5)	15 (31.3)
Ki67 (%)	42.6	30.6	33.1	37.2

### Flow cytometry

Tumor single cells and blood samples were stained with fluorochrome labeled antibodies (clone and distributor information as well as panel design are summarized in Suppl. Table 1). Samples were incubated for 30 min at 4 °C and subsequently washed twice (300 x g, 5 min, 4 °C) with DPBS containing 1% FBS and 0.01% NaN_3_. Appropriate immunoglobulin antibodies were used as isotype controls. Protein expression profiles of tumor and immune cells were analyzed by flow cytometry with a FACS-Canto-II (BD Biosciences, San Jose, CA, USA), which was run by the Diva software Ver. 7.0 (BD Biosciences, San Jose, CA, USA). Results were analyzed using the FlowJo software v10.8 (BD Biosciences, San Jose, CA, USA).

### Soluble factor analyzes by legendplex™ bead-based immunoassay

Plasma was collected via centrifugation of peripheral EDTA blood (2000xg for ten minutes at 4 °C) and stored at -80 °C. Multiplex assay procedure of LEGENDplex™ 12-plex HU Immune Checkpoint Panel 1 (Cat No. 740867, analyzed molecules: sCD25, 4-1BB, sCD27, B7.2, free active TGF-ß1, sCTLA-4, sPD-L1, sPD-L2, sPD-1, sTIM-3, sLAG-3, and sGalectin-9) was performed according to manufacturer´s protocol. Briefly, human plasma samples were pre-diluted and were incubated with microbeads (800 rpm; 2 h, room temperature (RT)), and after several washing steps incubated with detection antibody (800 rpm, 1 h, RT) followed by Streptavidin-PE (SA-PE) incubation (800 rpm, 30 min, RT). Data were analyzed with the LEGENDplex™ Data Analysis Software Suite.

### Literature based TNBC patient scoring

Based on the literature, all analyzed markers were categorized as beneficial or unfavorable (Table 2) and samples with values above the calculated mean in the analyzed TNBC cohort were allocated to the value of + 2 or -2, respectively. Each marker was equally weighted because at the current state there is no reasonable evidence for a different weighting. Scores occurring on both T cell subsets (CD4 or CD8) were divided into values of + 1 or -1. Two TNBC patients with multiple missing biomarker information (e.g., small tumor tissue, no blood sample) and two patients without ICI treatment or additional (trastuzumab) treatment due to the co-existence of another tumor subtype were excluded. All scores for the individual markers were summed up to a final score and results < 0 was considered to predict residual disease (pre-RD) and ≥ 0 were considered to predict pCR (pre-pCR).

**Table 2:** Exemplary literature for all markers included in the predictive score model. Negatively associated markers are typed in red and positive categorized markers in green

Biomarker	Prognostic/predictive value and references
**mMHC-I**	• MHC-I loss associated with a lack of response to immunotherapy [19, 20]
**TILs**	• Pos. prognostic factor in TNBC [21, 22] • Predictive in atezolizumab [23] and pembrolizumab [24] treated mTNBC
**mPD-1**	• Pos. prognostic factor for survival in many cancer subtypes including BC [25]
**mCD137**	• Costimulatory receptor expressed on activated T lymphocytes, dendritic cells (DCs), and NK cells [26] • Predictive for pembrolizumab treated HNSCC patients [28]
**CM/EM T cells**	• High numbers of CD45RO^+^ memory T cells within different primary malignancies [29] including TNBC [30] are associated with favourable clinical outcome.
**CD8 in TIL**	• Higher CD3^+^ infiltration [31] as well as CD8^+^ infiltration [32] associated with increased pCR in BC patients receiving NAT • Positive predictive for immunotherapy response in different cancers [33–35] including BC [35, 36] • Predictive for improved progression free survival (PFS) and OS after treatment with atezolizumab in TNBC [37]
**mPD-L1 on myeloid cells**	• Associated with good clinical outcome in BC [38, 40] • Predictive for response to *n*eoadjuvant Durvalumab treatment in TNBC [41]
**LAG-3+** ** TIL**	• Associated with better OS in different types of cancers [14] also in TNBC [13, 42] and ER^-^ [42, 43]
**TIM-3+** ** TIL**	• Independent favorable prognostic factor in BC [11, 44–46] • Increased levels associated with improved survival in BC treated with chemotherapy [47]
**sLAG-3**	• Prognostic (OS, PFS) in mHR^+^ [48] • High concentration before ICI in solid cancer significantly impaired PFS and OS [51]
**MHC II**	• High MHC-II expression associated with positive responses to IC therapy in melanoma [52, 53] [54] and in HER2^-^ BC [55]. • Associated with an improved prognosis in TNBC [56–59]
**CD24**	• Associated with poor prognosis, tumor size and lymph node positivity in BC [60]
**mPD-L1 (tumoral)**	• Associated with a worse OS in BC [61]
**sPD-1**	• Associated with advanced disease and worse outcome [62] • High levels have been associated with poor cytotoxic therapy response in TNBC [63]
**sPD-L1**	• More likely to develop progressive disease upon ICI treatment in different solid tumors [64], specifically in lung [65] and melanoma patients [66, 67].
**sPD-L2**	• Higher sPD-L2 levels in patients with Ki67 > 30% and in tumor grade III-IV BC patients [68].
**sCD25**	• Negative prognostic marker in HNSCC [70, 71], lung [72], and multiple myeloma [73] patients. • Lack of long term benefit of ICI in lung [72] and association with resistance to CTLA-4 blockade in melanoma [74]
**sCD27**	• Negative prognostic factor associated with reduced survival in lung cancer [75], HNSCC [70], and diffuse large B-cell lymphoma [76] • Negative prognostic marker in solid cancer patients undergoing ICI [51]
**sTIM-3**	• Associated with advanced NSCLC disease [77] or increased invasion [78], and anti-PD-1 resistance [79] • Low sTIM-3 level correlated to increased response to anti-PD-1 treatment [80] in NSCLC patients
**Gal-9**	• Soluble immunosuppressive agents in various malignancies [81, 82].
**CD33 in PB**	• High pre-treatment myeloid cells were associated with lower pathologic complete response (pCR) rates to neoadj. Chemotherapy in TNBC [83].
**PD-L1 on B cells**	• PD-L1 expression on B cells pursue significant immunosuppressive effect in various tumors [86]

## Statistical analyses

The results are shown as mean and standard deviation (SD), as described in the figure legends. Statistical analyses were performed using the GraphPad PRISM 8. We used a significance level of *p* ≤ 0.05 in the one-way ANOVA with Tukey’s multiple comparisons test, two-way ANOVA, and paired parametric t-test, or if data were non-parametric Kruskal-Wallis test with Dunn´s multiple comparisons test. Correlations were determined using a two-tailed Pearson correlation test. Applied test information is included in the figure legend and asterisks denote statistical significance (* *p* ≤ 0.05, ** *p* ≤ 0.01, *** *p* ≤ 0.001; **** *p* ≤ 0.0001).

### Ethics statement

Patient-derived tumor samples and peripheral blood samples were taken with approval from the ethics committee of the University of Regensburg (BC samples and blood: 22-3151-101, Changes and adjustments – blood: 22-3151_1-101). All patients have signed a written informed consent.

## Results

### Individual MHC-I, -II, CD24, CD44, and PD-L1 expression profiles detected in BC tissues

**Fig. 1:**
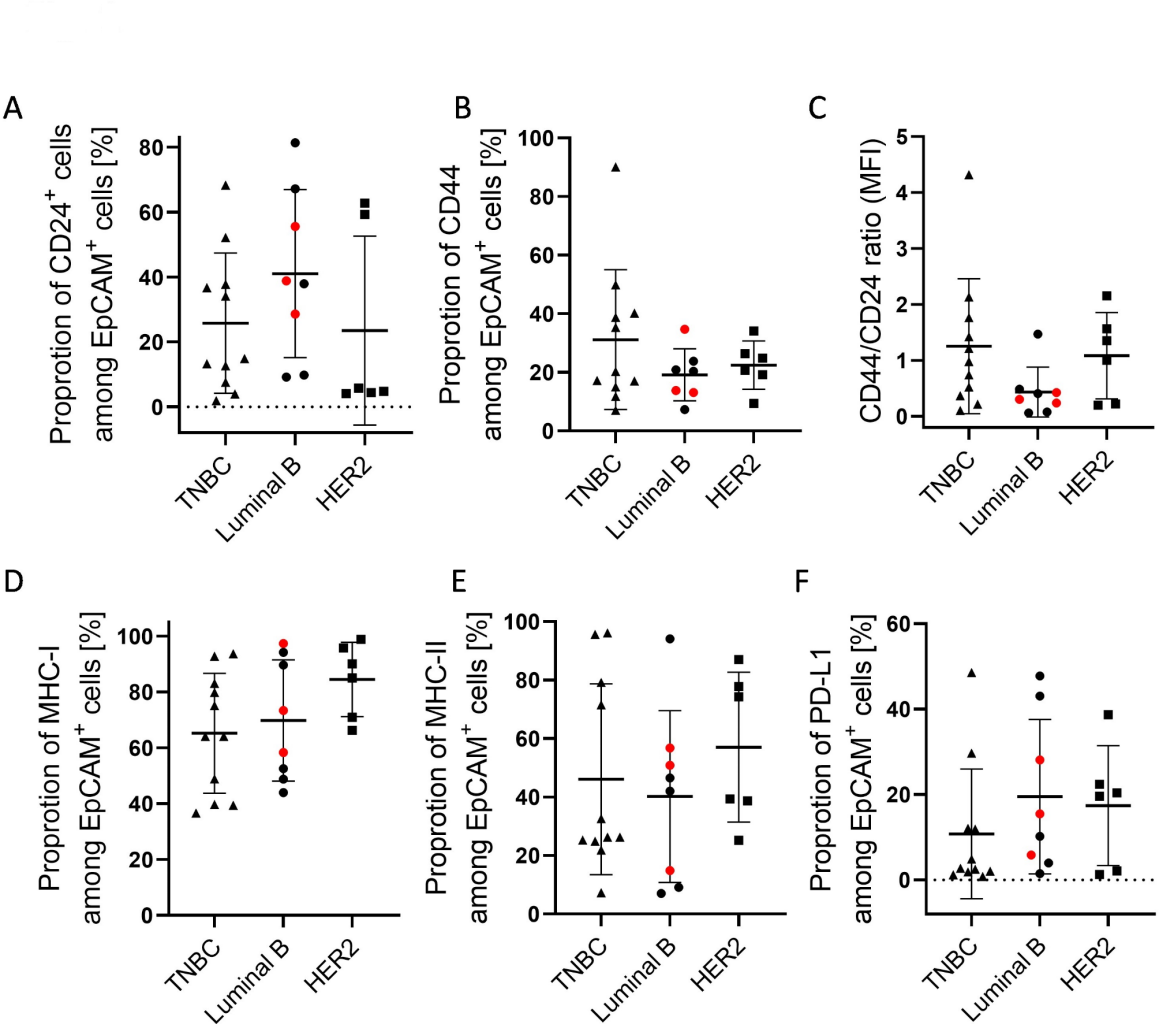
Tumor cell characterization in BC subtypes (TNBC, Luminal B, HER2^+^). EpCAM^+^ tumor cells were analyzed by flow cytometry. The percentage of CD24^+^ (**A**) or CD44^+^ tumor cells (**B**) and the CD44/CD24 ratio (**C**) of mean fluorescence intensities (MFI) are displayed. Graphs represent the proportion of MHC-I (**D**), MHC-II (**E**) and PD-L1 (**F**) among EpCAM^+^ tumor cells. Each symbol represents a single donor; Data are given as mean ± SD (no significnt differences were detected by Tukey’s multiple comparisons test). Red symbols represent Luminal B breast cancer patients with HER2 over-expression

Tumor expression profiles considering BC subtypes were investigated by flow cytometry. Epithelial cell adhesion molecule (EpCAM) was used to identify tumor cells in BC samples. In all tested tumor samples, the expression profile of CD24, PD-L1, and MHC-I & II showed a broad spectrum of expression in the range between 0->90% in all tested BC subentities (Fig. 1). The CD24 (Fig. 1A) and CD44 positivity (Fig. 1B) and thus CD44/CD24 ratio (Fig. 1C) varied especially in TNBC whereas Luminal B and HER2^+^ tumors showed rather low CD44 expression. In the context of MHC-I, the majority of tumor cells expressed this antigen-presenting receptor except for three TNBC samples with expression levels below <40% (Fig. 1D). MHC-II expression varied in all tested BC samples in a range of 7 to 97% (Fig. 1E). The lowest average of PD-L1 expression was found in TNBC (Fig. 1F).

### T cells are the main immune cell population among tumor-infiltrating lymphocytes

**Fig. 2:**
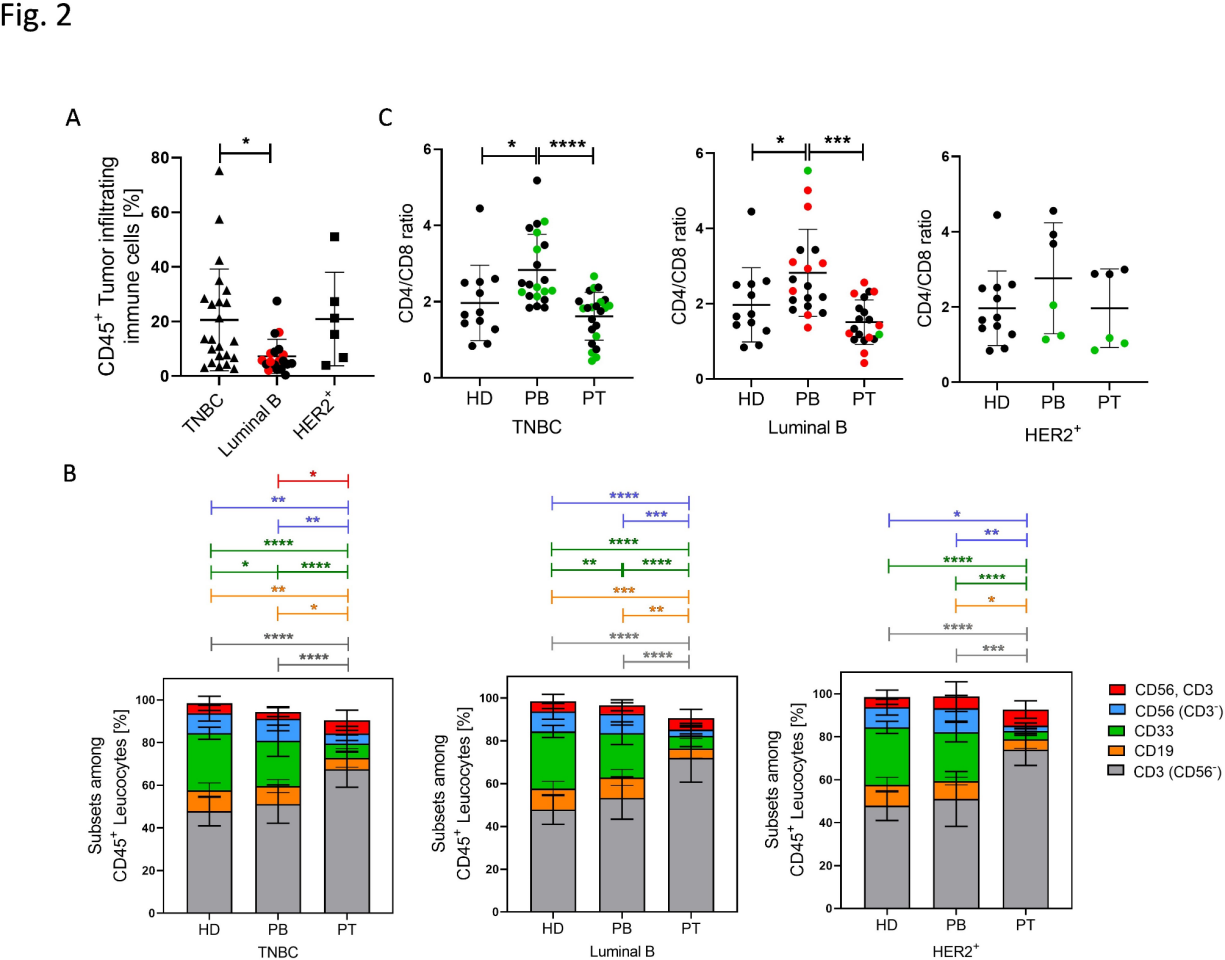
Flow cytometric analysis of immune cells in blood of healthy donors and BC patient samples. (**A**) Immune cell infiltration in the tumor tissue are displayed. (**B**) Mean proportions of T lymphocytes (grey), B lymphocytes (orange), myeloid cells (green), NK cells (blue), NK-T cells (red) in blood and tumor samples are displayed. (**C**) CD4^+^ and CD8^+^ T cell proportion of CD3^+^ T cells are given for TNBC, Luminal B, HER2^+^ in comparison to healthy donors. Data are shown as mean +/-SD and p-values were calculated by Kruskal-Wallis-test (Dunn´s multiple comparisons test) or one-way ANOVA (Tukey´s multiple comparisons test) based on parametric pretesting; * *p* ≤ 0.05; ** *p* ≤ 0.01; *** *p* ≤ 0.001; **** *p* ≤ 0.0001; HD = peripheral blood of healthy donors; PB = patient derived peripheral blood; PT = patient derived tumor

The average of immune cell infiltration in the tumor tissue in TNBC and HER2^+^ analyzed by flow cytometry was ~ 20% and the lowest infiltration was seen in Luminal B tumors (Fig. 2A). The majority of tumor infiltrating immune cells belong to the T cell subsets in all tested cancer subtypes (Fig. 2B). In the peripheral blood (PB) of TNBC and Luminal B patients the ratio of CD4/CD8 was significantly increased compared to the healthy donor samples and the tumor tissue (Fig. 2C). Overall, there were no significant differences in immune cell distribution between the healthy donor and patient blood and in between tumor entities detectable.

**Fig. 3:**
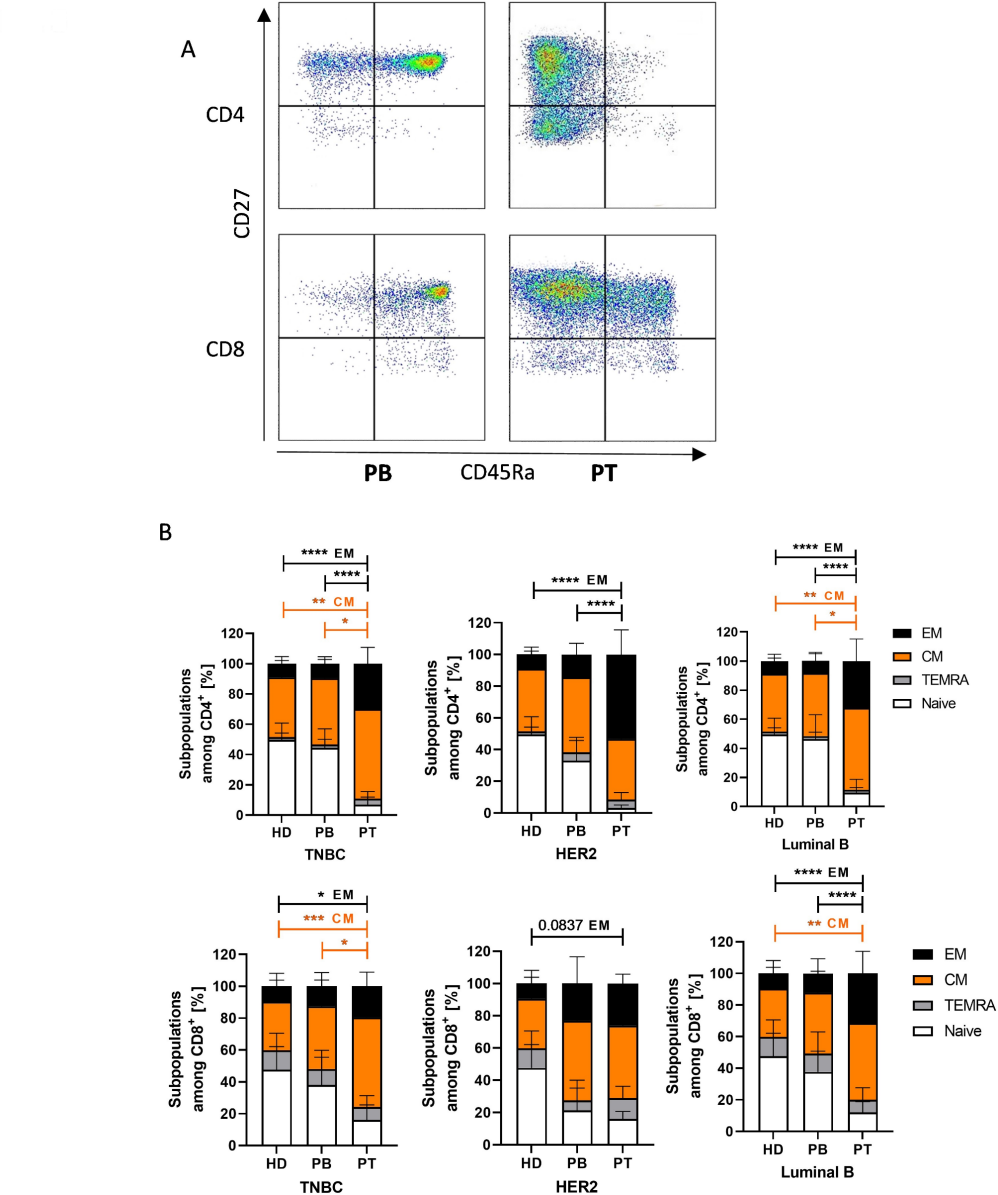
T cell maturation status in the blood and tumor tumor tissue were quantified. (**A**) Gating strategy using CD45RA and CD27 expression are shown to determine effector memory (EM) (CD45RA^-^, CD27^-^), central memory (CM) (CD45RA^-^, CD27^+^), naïve (CD45RA^+^, CD27^+^) and terminal differentiated EM (TEMRA; CD45RA^+^, CD27^-^) on CD4^+^ and CD8^+^ T cells. (**B**). Maturation of CD4 (second row) and CD8 (third row) T cells were analyzed by flow cytometry in the peripheral blood of healthy donors (HD) and blood and tumor of TNBC, HER2 and Luminal B patients. Data are given as mean ± SD and significances calculated using Tukey’s multiple comparisons test. * *p* ≤ 0.05; ** *p* ≤ 0.01; *** *p* ≤ 0.001; **** *p* ≤ 0.0001

The mean percentage of CD137 expression on CD4 or CD8 T cells was ~ 10% without significant differences between blood and tumor or between blood from healthy donors and patients (Suppl. Figure 1 A&B). Only low concentration of soluble CD137 was detected (Suppl. Figure 1C). However, the expression of CD137 correlated significantly with LAG-3 (*p* < 0.0001) and TIM-3 (*p* = 0.037) on CD4^+^ T cells. CD137 on CD8^+^ T cells showed a correlation to the expression of PD-1 (*p* = 0.0034) and TIM-3 (*p* < 0.0001; Suppl. Figure 1D). Further phenotyping of tumor invading T cells revealed a central memory (CM) and end effector memory (EM) phenotype (Fig. 3A) in all entities. This cell fraction was significantly higher than in the PB of patients and healthy donors (HD) in TNBC and Luminal B patients (Fig. 3B). In HER2^+^ blood samples, the proportion of EM phenotype especially on CD8^+^ T cells was higher compared to other entities and therefore no significant differences between blood and tumor samples were measurable. Independent of subtypes, no significant changes in the blood of healthy donors or patients were detectable (Fig. 3B).

### Increased checkpoint expression on tumor-infiltratinglymphocytes 

**Fig. 4:**
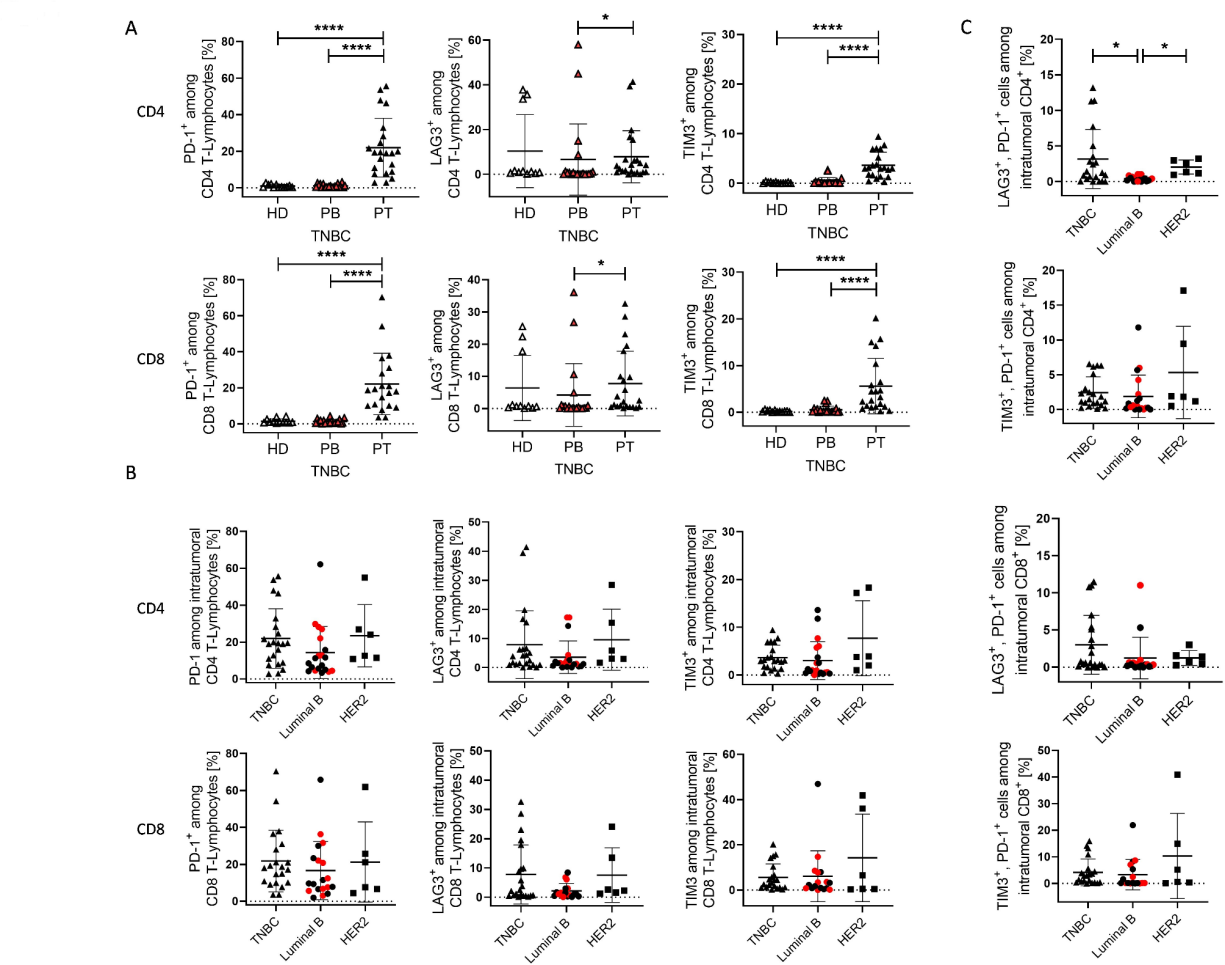
Checkpoint expression on T cells in the peripheral blood and tumor tissue. (**A**) Immune checkpoint marker (LAG-3, PD-1, TIM-3) expression was analyzed by flow cytometry in the peripheral blood of healthy donors and blood and tumor of TNBC patients. (**B**) Immune checkpoint marker (LAG-3, PD-1, TIM-3) expression (**B**) and co-expression (C) of BC patients (TNBC, Luminal B, HER2^+^) on CD4^+^ and CD8^+^ T cells are displayed. Data are shown as mean +/-SD and p-values were calculated by Kruskal-Wallis-test (Dunn´s multiple comparisons test); * *p* ≤ 0.05; **** *p* ≤ 0.0001; red symbols represent Luminal B BC patients with HER2 expression. HD = peripheral blood of healthy donors; PB = patient derived peripheral blood; PT = patient derived tumor

In TNBC samples, the expression of PD-1 and TIM-3 on CD4^+^ and CD8^+^ T cells was significantly higher in the tumor tissue compared to the peripheral blood (Fig. 4A). Interestingly, in the blood samples of three healthy donors and two TNBC patients, a pronounced LAG-3 expression was detected (Fig. 4A). However, the mean percentage and the maximum of PD-1 expression on CD4^+^ and CD8^+^ T cells were higher compared to LAG-3 and TIM-3 (Fig. 4B).

Comparison between BC subtypes revealed no significant differences in checkpoint expression on CD4^+^ or CD8^+^ T cells (Fig. 4B). A significantly increased proportion of tumor infiltrating CD4^+^ T cells in TNBC (mean 3.16 +/- 4.16 SD; *p* = 0.05) and HER2^+^ (mean 2.03 +/- 0.99 SD; *p* = 0.018) patients showed a LAG-3/PD-1 co-expression compared to Luminal B patients (Fig. 4C). Even though the overall expression of PD-1 was low in the PB of patients, there was a correlation between expression in the peripheral blood and the tumor tissue (Suppl. Figure 2 A). A significant correlation was also detectable with respect to LAG-3 expression in the tumor and the PB (Suppl. Figure 2 A). The analysis of TIM-3 did not reveal such a correlation (data not shown). An additional correlation was found between PD-1 and TIM-3 expression on CD4^+^ and CD8^+^ T cells (Suppl. Figure 2B).

### High expression of PD-L1 detectable on myeloid and NK-T cells in the tumor tissue

**Fig. 5:**
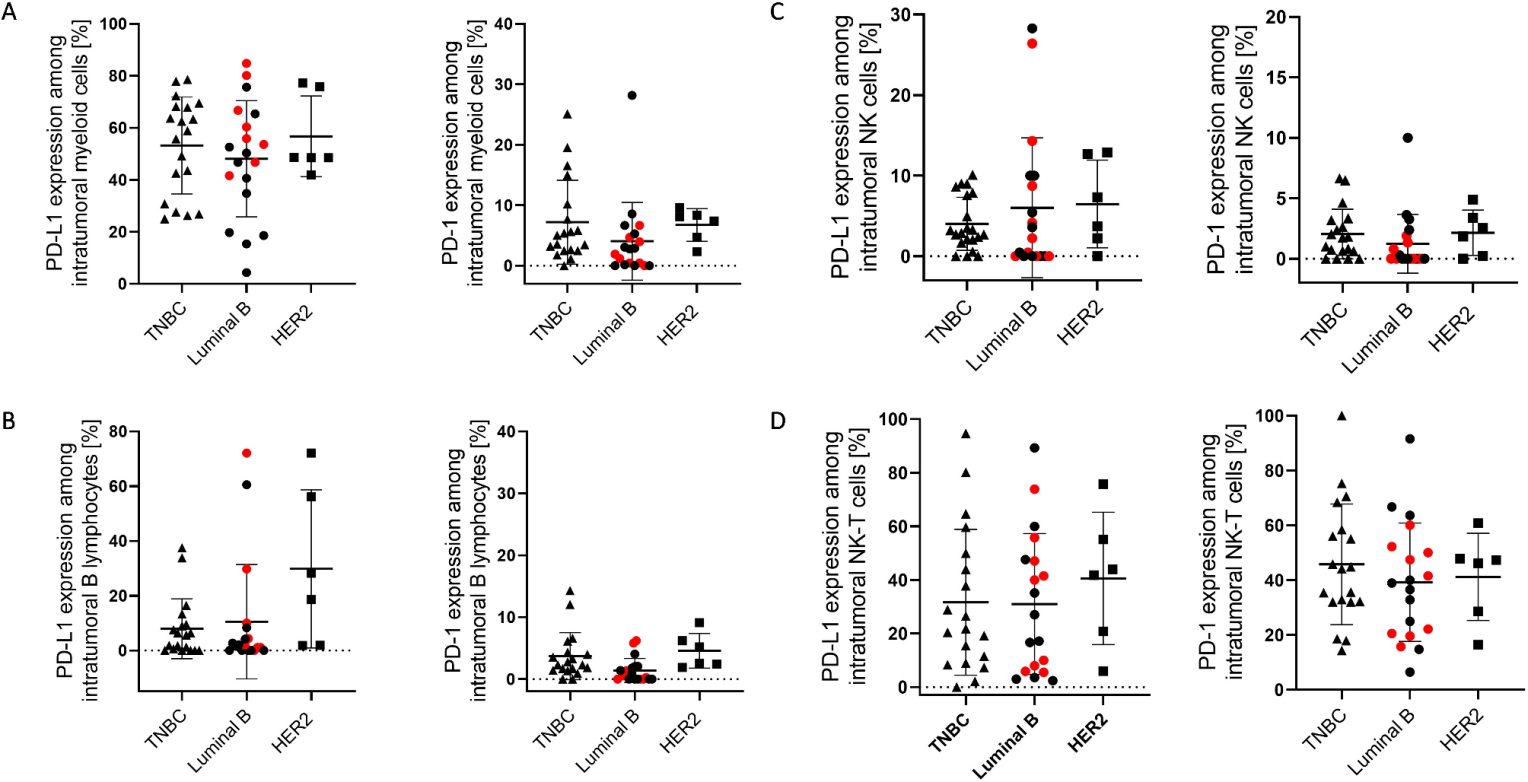
Flow cytometry analysis of PD-1 and PD-L1 expression on immune cell subsets of BC patients. Percentage of PD-1 and PD-L1 expression on intratumoral myeloid cells (**A**) and B cells (**B**), NK (**C**), and NKT (**D**) cells are displayed. Data are given as mean ± SD (no significant differences were detected by Tukey’s multiple comparisons test). Red symbols represent Luminal B breast cancer patients with HER2 over-expression

Intratumoral CD33^*+*^ myeloid cells expressed high levels of PD-L1 (average ~ 50%) but only a minority of cells indicated a PD-1 expression (Fig. 5A). Larger fractions of PD-L1 (but not PD-1) expression on B cells were detected in tumor tissues of some patients (Fig. 5B). PD-L1 and PD-1 expression on NK cells was low (Fig. 5C), whereas NK-T cells showed a wide range (0–94%) of PD-L1 as well as PD-1 (1-100%) appearance (Fig. 5D). No significant differences between BC subtypes were found.

### Broad range of soluble factors in individual BC patients

**Fig. 6:**
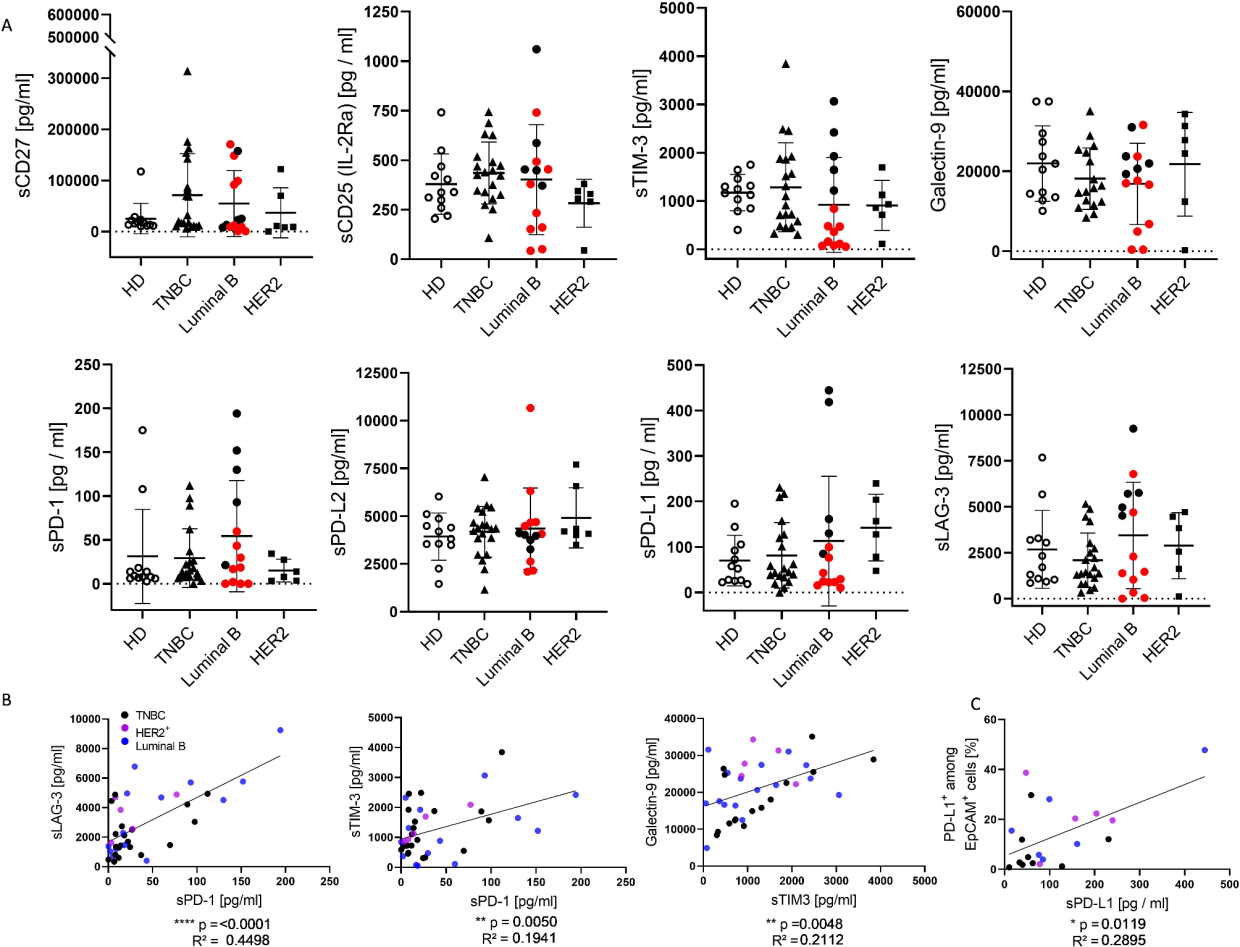
Soluble checkpoints and regulatory factors in plasma of BC patients compared to healthy controls. (**A**) Concentration of soluble factors (sCD27, sCD25) and soluble checkpoint molecules (sTIM-3, Galectin-9, sPD-1, sPD-L1 & 2, sLAG-3) were analyzed in plasma of cancer patients and healthy donors. Red symbols represent Luminal B breast cancer patients with HER2 over-expression. Data are given as mean ± SD (no significant differences were detected by one-way-ANOVA (Tukey’s multiple comparisons test). (**B**) Correlations of sLAG-3 and sTIM-3 to sPD-1 and between Galectin-9 and sTIM-3 of breast cancer patients (each entity represented by different color) are displayed. (**C**) Correlation of PD-L1 expression and secretion were determined. Correlation were determined using the two-tailed Pearson correlation test and p-values are indicated in each graph

Overall, a diverse concentration of soluble factors was found in the plasma but without significant differences to healthy controls (Fig. 6A). The concentration of sCD27, sTIM-3, Gal-9, and sLAG-3 varied remarkably between individuals. Interestingly, in some plasma samples derived from the so-called low immunogenic HER2^-^ Luminal B tumors, high concentrations of sTIM-3, sPD-1, sPD-L1, and sLAG3 were found. In contrast to PD-L2 and Galectine-9 only low concentrations of PD-1 and PD-L1 were counted. We also detected a profound correlation between sLAG-3 and sPD-1 and a moderate correlation between sTIM-3 and sPD-1 as well as Gal-9 and its binding partner sTIM-3 (Fig. 6B). There were no additional correlation detectable between the analyzed soluble checkpoint molecules. Furthermore, the soluble PD-L1 levels correlated with the frequency of PD-L1 expression on tumor cells (Fig. 6C).

### Scoring of membrane bound and soluble factors allowed the prediction of response to immunotherapy in TNBC patients

Due to the wide range of detected expression and secretion profiles, the analyzed markers were scored positive (+ 2 points) and negative (-2 points) based on the literature (Table 2). The total score summed up to the “predictive score” (Table 3), which we assessed for each patient (Suppl. Table 2). Next we assigned patients with values below 0 to the group with prediction for residual disease (pre-RD) and values ≥ 0 to the group with prediction for pCR (pre-pCR) Non-responders were not observed in this study. These results were assigned to the primary endpoint data (pCR, non-pCR at the time point of surgery; Table 3). In this small group of patients, it accurately predicted treatment outcomes for fifteen patients but was incorrect for four patients, resulting in an accuracy of 79%. This study revealed a sensitivity of 83.3%, a specificity of 76.9%, a positive predictive value of 62.5%, and a negative predictive value of 90.9% for therapy response prediction.

Notably, focusing on the TIL rate only did not reveal (potential) responders. More specifically, seven patients had higher TIL levels (Suppl. Table 2) but only three achieved pCR at the time of surgery (Table 3). Additionally, three patients with pCR did not exhibit increased immune cell infiltration.

**Table 3:** Scoring of 19 TNBC patients (time point of diagnosis) based on membrane bound and secreted markers (Suppl. Table 1) correlated with the primary clinical endpoint (pathological complete response (pCR) or non-pCR; time point of surgery). Prediction for pCR (pre-pCR; total score ≥ 0); Prediction for residual disease (pre-RD); total score < 0); match = predicted outcome based on the score is in accordance with the outcome determined by the pathologist; *mismatch* = predicted outcome based on the score is not in accordance with pathological outcome (*italic*)

# of TNBC patient	beneficial factor score	detrimental factor score	total score	prediction of score	pathological outcome data	match/ mismatch
#1	19	-10	9	*pre-pCR*	pCR, tumor free	match
#2	7	-6	1	*pre-pCR*	pCR, tumor free	match
#3	4	-6	-2	*pre-RD*	non-pCR	match
#4	21	-6	15	*pre-pCR*	pCR, tumor free	match
#5	4	-10	-6	*pre-RD*	non-pCR	match
#6	6	-10	-4	*pre-RD*	non-pCR	match
#7	6	-6	0	*pre-pCR*	pCR, tumor free	match
#8	*8*	*-4*	*4*	*pre-pCR*	non-pCR	*mismatch*
#9	1	-8	-7	*pre-RD*	non-pCR	match
#10	3	-14	-11	*pre-RD*	non-pCR	match
#11	*12*	*-10*	*2*	*pre-pCR*	non-pCR	*mismatch*
#12	*17*	*-10*	7	*pre-pCR*	non-pCR	*mismatch*
#13	3	-4	-1	*pre-RD*	non-pCR	match
#14	5	-8	-3	*pre-RD*	non-pCR	match
#15	*12*	*-14*	-2	*pre-RD*	pCR, tumor free	*mismatch*
#16	3	0	3	*pre-pCR*	pCR, tumor free	match
#17	1	-8	-7	*pre-RD*	non-pCR	match
#18	4	-10	-6	*pre-RD*	non-pCR	match
#19	7	-10	-3	*pre-RD*	non-pCR	match

## Discussion

ICIs are promising and in some instances, powerful therapeutic options for the treatment of different cancer subtypes with a still growing number of ongoing clinical trials. TNBC is considered to represent an immunogenic BC subtype, mainly due to a greater tumor mutational burden (TMB) and a pronounced immune cell infiltration, which assures pronounced susceptibility to immunotherapeutics. However, only a subgroup of TNBC patients respond to ICI therapy [87]. Thus, the identification of predictive biomarkers for single and multiplexed immune checkpoint treatments is urgently needed. Here, we quantitatively determined soluble and membrane-located markers at the time of BC diagnosis in different BC subtypes. Markers evaluated in TNBC patients, who were eligible for a checkpoint treatment, were rated based on the literature and summarized to a predictive score (≥ 0 = “complete responder” with predicted pCR, < 0 = “partial responder” with predicted residual disease). At the end of neoadjuvant treatment, pCR was determined and revealed 79% accuracy of prediction.

Considering all tested BCs, regardless of subtypes, PD-1 was the most dominant checkpoint on T cells, which confirms its potency to serve as checkpoint therapy. Limited quantitative data are available comparing the expression profile of PD-1, LAG-3, and TIM-3 in early BC. Mollavelioglu and colleagues analyzed BC tumor tissues by a quantitative and correlative flow cytometric approach. They found co-expression of PD-1, LAG-3, TIM-3, LAG-3 and CTLA-4 on TILs in early stage BC samples and increased presence of LAG-3, PD-1, and TIGIT on CD8^+^ TIL in T2 tumors [88]. However, the majority of samples in their study (*n* = 26) was attributed to the luminal group and only three TNBC and three HER2^+^ were included. We also identified varying levels of PD-1 expressions that indicates the need for individualized alternative (checkpoint) therapies, especially in those patients with increased co-expression of LAG-3 and TIM-3. Interestingly, we detected not only the correlation between expressed but also between different secreted checkpoint molecules, which confirms co-occurrence and the potential of dual targeting. Co-expression of checkpoint molecules has been described before, e.g., in TNBC patients [7, 88]. More specifically, Du and colleagues reported enhanced anti-tumor efficiency in a TNBC based mouse model treated with LAG3 and PD-1 dual blockade [7]. Based on the RELATIVITY-047 trial, first combination strategies for PD-1 (nivolumab) and LAG-3 (relatlimab) targeting have been approved by the FDA for the treatment of unresectable or metastatic melanoma. The median PFS of patients treated with combination was doubled compared to the PFS of patients who underwent nivolumab monotherapy only [9]. However, further evaluation of the potential benefit in patients is needed. Soluble checkpoint variants might also affect the efficiency of non-combined ICI. Therefore, we included the soluble variants in our scoring model. Some of the soluble factors have been associated to prognosis and prediction in different cancers and are summarized in Table 2. For instance, sCD27 is a negative prognostic factor in solid cancer patients undergoing ICI treatment [51]. As reported before, soluble PD-1 concentration was low and only HER2 negative Luminal B patients showed slightly enhanced levels in the plasma. It has been postulated that high sPD-1 levels before treatment are associated with advanced disease and worse outcome, whereas increasing sPD-1 levels upon treatment (including checkpoint therapy) have been linked to improved PFS and OS [62]. Several publication report on divergent concentration of soluble markers in the PB of cancer patients compared to samples derived from healthy donors, even though the reports are inconsistent. However, in agreement with our data, marker concentrations of sPD-L1 in TNBC [63] or sPD-1 and sLAG-3 in early BC patients of all subtypes [89] appeared comparable to healthy donors without significant differences. Just recently, the impact of soluble factors in various cancers was summarized in an article, emphasizing that further investigation is needed to better understand their role in diagnosis, prognosis, and therapy response [16].

Interestingly, in the HR^+^ subtype, HER2 status divided patients into two clusters with noticeably higher concentration of sTIM-3, sPD-1, sLAG3, and sPD-L1 in HER2-negative tumors. This indicates an immune suppressive situation in HR^+^HER2^-^ rather than a low immunogenic disease. Accordingly, an increased infiltration of tumor associated macrophages and the presence of non-activated cancer associated fibroblasts in HR^+^ tumors have been observed, which represents an immunosuppressive environment as well [90]. In line with this assumption, clinical trials done on high-risk ER^+^ BC patients with combined NACT and ICI achieved significantly improved pCR rates compared to the control arm [91].

Currently, PD-L1 assessed by immunohistochemistry (IHC) is the only validated predictive marker for ICIs in TNBC. In addition, in TNBC PD-L1 expression (> 1% on immune cells or > 10% combined positive score) is predictive for ICI response in the advanced [3, 5] but not in early diseases [2, 6]. Other predictors for therapy responses are microsatellite instability [92] and the presence of high TMB [93–95] but only a small percentage of TNBC could be rated based on these categories [96].

Increased infiltration of TILs have also been associated with an increased response rate to NACT [21] and ICI [37, 94, 95, 97, 98]. However, on a case-by-case basis, the composition of infiltrating immune cells considerably varies and determines the immune activity both in the absence and presence of ICI treatments. In the 19 TNBC cases analyzed in our study, the TIL rate was not predictive of ICI response, as four patients showed increased infiltration but did not achieve pCR, while three patients with pCR did not display elevated immune cell infiltration.

Therefore, the multifaceted immune status of the individual patients needs to be integrated into a valid, diagnostic assay. First steps have already been done for example by the design of multiplex assays that allow an image based immune profiling to identify predictive and prognostic (immune) signatures in BC patients (summarized in [99]). Yin and colleagues stratified BC patients into high and low risk cancers based on a seven gene expression assay (BATF, CD3D, HLA-DQB2, JUN, MAP2K6, NFKBIE, PAK1). Patients with high-risk tumors showed reduced overall survival and significant differences depending on the clinic-pathological parameters and immune cell infiltration rate. The authors applied this profile-derived nomogram and thereby predicted drug susceptibility and immune response [100]. Denkert and colleagues analyzed gene expression in 247 biopsies (pretreated and during treatment cycles) based on the GeparNuevo trial [101]. Triggered by one cycle of durvalumab, an increased immune activation and reduced expression of proliferation associated genes was observed. Regardless of the therapy applied, immune related genes turned out as positive prognostic factors, whereas once again PD-L1 was identified as the most significant prognosticator for distant disease-free survival.

Hammerl and colleagues analyzed 681 samples using multiplexed immunofluorescence, conventional IHC, gene expression, and TCR clonality assays. They classified tumors into three spatial immunophenotypes (“excluded”, “ignored”, and “inflamed”) based on TILs and CD8^+^ T cell infiltration. TNBC tumors with increased CD8^+^ T cell infiltration (“inflamed”) before treatment showed the best response to ICI treatment and were characterized by high TCR clonality and PD-1, TIM-3, and LAG-3 co-expression. Resistance was, inter alia, associated with increased glycolysis (“excluded phenotype”) or CD163 myeloid cell infiltration (“ignored phenotype”) [102].

Single cell analysis with imaging mass cytometry of samples from the neoTRIPaPDL1 clinical trial allowed the characterization of specific tumor microenvironment phenotypes, and revealed a predictive role of PD-L1^+^ indoleamine 2,3-dioxigenase^+^ APCs and CD56^+^ neuroendocrine epithelial cells [103]. RNA-seq data from 242 patients of this trial were also analyzed by the determaIO assay, which includes a 27-gene signature that characterizes the phenotype of the tumor immune microenvironment. The authors found an enhanced probability of achieving pCR upon ICI treatment in patients with DetermaIO-positive tumors [104].

There are only a few studies that use flow cytometry to assess the prognostic or predictive value in BC patients. Cattin observed an increased frequency of CD117^+^CD11b^+^ granulocytes and regulatory T cells in the PB associated with radiotherapy, but this study included only 13 patients and did not differentiate between BC subtypes [105]. In a cohort of 51 advanced BC patients, flow cytometry data from PB revealed an increase in activated OX40^+^/PD-1^−^ T lymphocytes and a decrease in inhibitory myeloid cells and Tregs, which correlated with the clinical benefit upon systemic treatment [106]. Dyikanov and colleagues developed a platform that integrates flow cytometry-based immunophenotyping with bulk RNA-sequencing data [107]. They found an increased number of CX3CR1^neg^ CD8^+^ TEMRA cells and monocytes, along with a decrease in naïve and memory B cells in patients compared to healthy donors. However, they combined data from various solid tumors (mostly pre-treated) and found that flow cytometric data generated from patients with similar diagnoses or treatments did not form distinguishable clusters.

All these various approaches have been applied to define a predictive or prognostic signature and underline the need for multiplexed immune-profiling. Here, we tested different secreted and expressed molecules by flow cytometry and LegendPlex assay and integrated the data into a scoring model with a potentially predictive value and an accuracy of 79%. This scoring system can be further adapted by selection of most relevant markers to further enhance the accuracy and the predictive power. This first proof-of-principle concept of applying a scoring system represents a promising approach to identifying potential responders to checkpoint therapy in TNBC, highlighting the importance of individualized treatment strategies based on specific immunological profiles. However, only 19 TNBC patients, who received ICI treatment in accordance to the 522 trial [6] are included in this study. In addition, the scoring of all markers that is based on selected, relevant literature is of retrospective nature. Thus, further prospective clinical studies including a higher number of patients and additional adjustments (e.g., adapted marker selection, specified pre-defined cut-offs) are required to validate the evidence for clinical applications. Additionally, long-term follow-up is essential to assess not only the early response to ICI (pCR at the time of surgery) but also sustained long-term remission.

## Conclusions

Overall, PD-1 was the highest expressed checkpoint molecule confirming its great potential for ICI. However, in individual patients, other checkpoints such as LAG-3 and TIM-3 were considerably (co-) expressed. Scoring of relevant individual expression and secretion markers in ICI-treated TNBC patients before treatment enables to set-up a “predictive score”. This score was in accordance with the pCR determination in 79%. Possibly, an adapted scoring model for HR^+^ and HER2^+^ BC patients enables the identification of eligible patients within this subtypes likewise. Therefore, a scoring-based approach to summarize multiplexed marker profiling might be an option to distinguish those patients, who most likely will benefit from checkpoint mono-therapy from those patients, who require additional combination therapies.

## Electronic supplementary material

Below is the link to the electronic supplementary material.

Supplementary Material 1

Supplementary Material 2

## Data Availability

No datasets were generated or analyzed during the current study.

## Abbreviations

ADAM 10,17: Disintegrin and metalloproteinase domain-containing protein 10
APC: Antigen presenting cell
BC: Breast cancer
Breg: Regulatory B cell
CEACAM1: Carcinoembryionic antigen-related cell adhesion molecule 1
CM: Central memory
CPS: Combined PD-L1 positive score
CTLA-4: Cytotoxic T lymphocyte associated protein 4
DFS: Disease free survival
DPBS: Dulbecco´s phosphate buffered saline
EM: Effector memory
EMA: European Medicines Agency
EpCAM: Epithelial cell adhesion molecule
FDA: Food and Drug Administration
Gal-9: Galectin 9
HD: Peripheral blood of healthy donors
HER2: Human epidermal growth factor receptor 2
HMGB1: High mobility group protein B1
HR: Hormone receptor
ICI: Immune checkpoint inhibitor
LAG-3: Lymphocyte activation gene 3
MFI: Mean fluorescence intensity
MHC-I, -II: Major histocompatibility complex I, II
NACT: Neoadjuvant chemotherapy
NK cell: Natural killer cell
PB: Patient derived peripheral blood
pCR: Pathological complete responder / response
PD-(L)1, -L2: Programmed cell death (ligand) 1, ligand 2
PFS: Progression free survival
PT: Patient derived tumor
pre-pCR: Predict pCR
pre-RD: Predict residual disease
SD: Standard deviation
TCR: T cell antigen receptor
TEMRA: Terminally differentiated effector memory T cells
TGF-β: Transforming growth factor β
TIL: Tumor infiltrating lymphocytes
TIM-3: T cell immunoglobulin and mucin domain 3
TMB: Tumor mutational burden
TNBC: Triple negative breast cancer
Treg: Regulatory T cell
