# Supplementary material for: Breast cancer scoring based on a multiplexed profiling of soluble and cell-associated (immune) markers facilitates the prediction of pembrolizumab therapy
Source: Cancer Cell Int. 2025 Mar 27;25:120. doi: 10.1186/s12935-025-03729-7 (PMC11948714; doi:10.1186/s12935-025-03729-7)
Supplement: Supplementary file 1 — Supplementary Material 1 [file 12935_2025_3729_MOESM1_ESM.docx]

**Supplementary Figures and Tables**

**
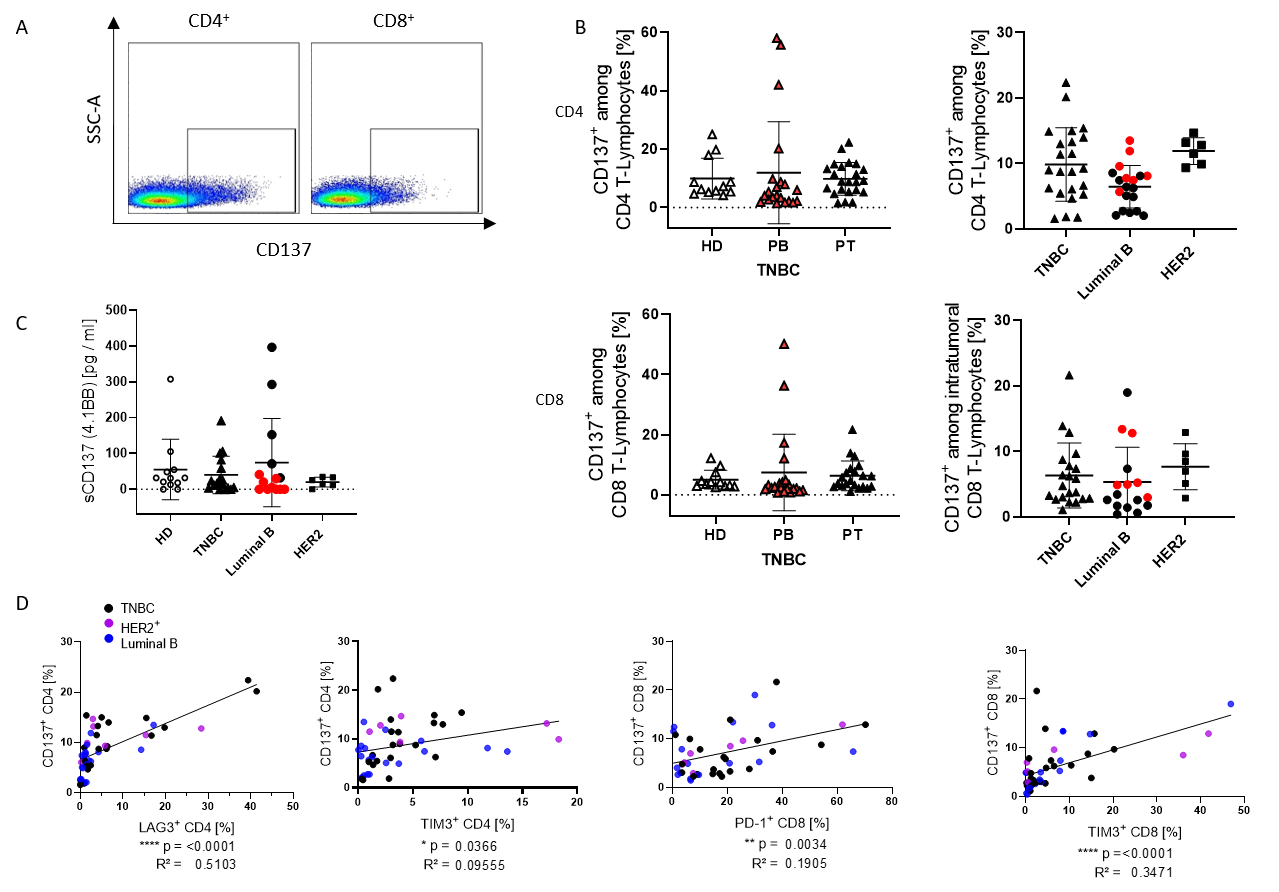
**

**Supplementary Figure 1:** CD137 expression and secretion in the peripheral blood and tumor tissue. (A) Exemplary gating strategy for CD137 expression on CD4+ and CD8+ T cells is shown. (B) CD137 expression on CD4 (first row) and CD8 (second row) were analyzed by flow cytometry in the peripheral blood of healthy donors and blood and tumor of BC patients. (C) Graph summarizes the concentration of soluble CD137 in the plasma of BC patients and healthy donors. Data are given as mean ± SD (no significances were detected using Tukey's multiple comparisons test). Red symbols represent HER2+ Luminal B breast cancer patients. (D) Correlations of CD137 and LAG-3, PD-1, and TIM-3 expression in the tumor of breast cancer patients are displayed (the entities are represented by different color). Correlation were determined using the two-tailed Pearson correlation test and statistic values are indicated in each graph.

**
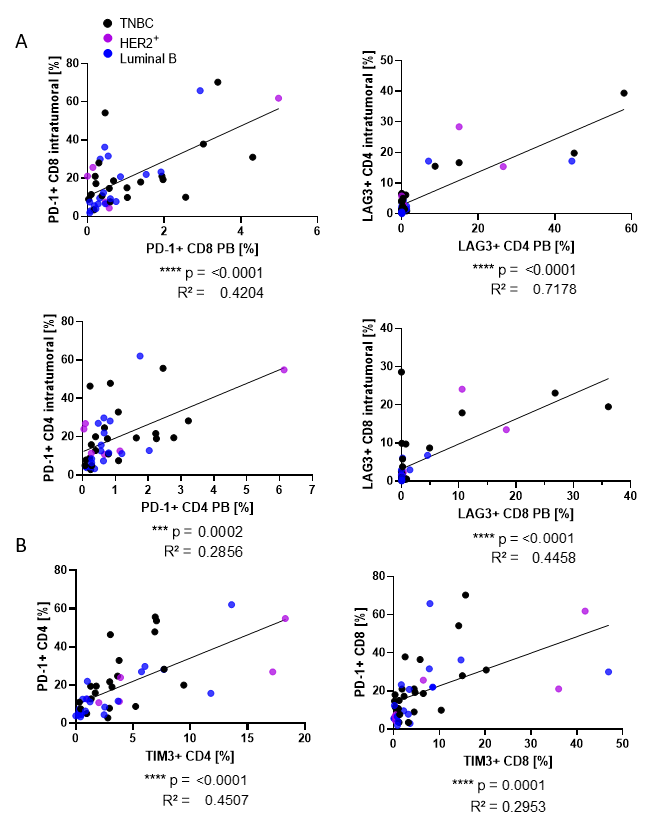
**

**Supplementary Figure 2**: Correlation between checkpoint expression in BC patients. (A) Correlation between PD-1 and LAG-3 expression on CD4+ and CD8+ T cells in peripheral blood associated to tumor infiltrating cells are displayed (each entity represented by different color). (B) Correlation between PD-1 and TIM-3 in tumor infiltrating CD4+ and CD8+ T cells are depicted. Correlation were determined using the two-tailed Pearson correlation  test. (D).

**Supplementary Table 1**: Panel design for the detection of membrane bound molecules on tumor and immune cells of breast cancer patients.

|  | Tumor cells | Tumor cells |  | TILs | Myeloid cells | T cells | T cells | T cells | T cells | T cells | T cells | Plasma |  |
| --- | --- | --- | --- | --- | --- | --- | --- | --- | --- | --- | --- | --- | --- |
| **# of TNBC patients** | **mMHC -I** | **mMHC-II** | **TILs** | **CD8 prop.** | **mPD-L1** | **CM** | **EM** | **mPD-1** | **mLAG-3** | **mTIM-3** | **mCD137** | **sLAG-3** | **Positive Value** |
| #1 | +2 | +2 | 0 | +2 | +2 | CD4: 0 | CD4: +1 | CD4: +1 | CD4: +1 | CD4: +1 | CD4: +1 | 0 | **19** |
|  |  |  |  |  |  | CD8: +1 | CD8: +1 | CD8: +1 | CD8: +1 | CD8: +1 | CD8: +1 |  |  |
| #2 | 0 | 0 | 0 | 0 | 0 | CD4: +1 | CD4: +1 | CD4: 0 | CD4: +1 | CD4: 0 | CD4: +1 | 0 | **7** |
|  |  |  |  |  |  | CD8: 0 | CD8: +1 | CD8: 0 | CD8: +1 | CD8: 0 | CD8: +1 |  |  |
| #3 | 0 | 0 | 0 | 0 | 0 | - | - | CD4: 0 | CD4: +1 | CD4: 0 | CD4: +1 | 0 | **4** |
|  |  |  |  |  |  |  |  | CD8: 0 | CD8: +1 | CD8: 0 | CD8: +1 |  |  |
| #4 | +2 | +2 | +2 | +2 | +2 | CD4: +1 | CD4: +1 | CD4: +1 | CD4: +1 | CD4: +1 | CD4: +1 | - | **21** |
|  |  |  |  |  |  | CD8: +1 | CD8: 0 | CD8: +1 | CD8: +1 | CD8: +1 | CD8: +1 |  |  |
| #5 | - | - | 0 | +2 | 0 | CD4: +1 | CD4: 0 | CD4: +1 | CD4: 0 | CD4: 0 | CD4: 0 | 0 | **4** |
|  |  |  |  |  |  | CD8: 0 | CD8: 0 | CD8: 0 | CD8: 0 | CD8: 0 | CD8: 0 |  |  |
| #6 | +2 | 0 | 0 | 0 | 0 | CD4: +1 | CD4: 0 | CD4: 0 | CD4: 0 | CD4: 0 | CD4: +1 | 0 | **6** |
|  |  |  |  |  |  | CD8: - | CD8: - | CD8: +1 | CD8: 0 | CD8: 0 | CD8: +1 |  |  |
| #7 | - | - | +2 | 0 | +2 | - | - | CD4: 0 | CD4: 0 | CD4: 0 | CD4: 0 | 0 | **6** |
|  |  |  |  |  |  |  |  | CD8: 0 | CD8: +1 | CD8: +1 | CD8: 0 |  |  |
| #8 | 0 | 0 | +2 | 0 | +2 | CD4: 0 | CD4: +1 | CD4: 0 | CD4: 0 | CD4: 0 | CD4: 0 | +2 | **8** |
|  |  |  |  |  |  | CD8: 0 | CD8: +1 | CD8: 0 | CD8: 0 | CD8: 0 | CD8: 0 |  |  |
| #9 | - | - | 0 | 0 | 0 | - | - | CD4: 0 | CD4: 0 | CD4: - | CD4: 0 | 0 | **1** |
|  |  |  |  |  |  |  |  | CD8: - | CD8: +1 | CD8: - | CD8: - |  |  |
| #10 | 0 | 0 | 0 | 0 | +2 | - | - | CD4: 0 | CD4: 0 | CD4: +1 | CD4: 0 | 0 | **3** |
|  |  |  |  |  |  |  |  | CD8: 0 | CD8: 0 | CD8: 0 | CD8: 0 |  |  |
| #11 | +2 | +2 | +2 | 0 | +2 | CD4: 0 | CD4: +1 | CD4: 0 | CD4: 0 | CD4: 0 | CD4: 0 | +2 | **12** |
|  |  |  |  |  |  | CD8: 0 | CD8: +1 | CD8: 0 | CD8: 0 | CD8: 0 | CD8: 0 |  |  |
| #12 | +2 | +2 | +2 | +2 | +2 | CD4: +1 | CD4: 0 | CD4: +1 | CD4: 0 | CD4: 0 | CD4: +1 | 0 | **17** |
|  |  |  |  |  |  | CD8: +1 | CD8: 0 | CD8: +1 | CD8: 0 | CD8: +1 | CD8: +1 |  |  |
| #13 | - | - | 0 | +2 | - | - | - | CD4: 0 | CD4: 0 | CD4: - | CD4: +1 | 0 | **3** |
|  |  |  |  |  |  |  |  | CD8: - | CD8: - | CD8: - | CD8: 0 |  |  |
| #14 | - | - | 0 | 0 | - | CD4: 0 | CD4: +1 | CD4: 0 | CD4: 0 | CD4: +1 | CD4: +1 | +2 | **5** |
|  |  |  |  |  |  | CD8: - | CD8: - | CD8: 0 | CD8: 0 | CD8: 0 | CD8: 0 |  |  |
| #15 | - | - | +2 | +2 | +2 | CD4: +1 | CD4: 0 | CD4: 0 | CD4: 0 | CD4: 0 | CD4: 0 | +2 | **12** |
|  |  |  |  |  |  | CD8: +1 | CD8: 0 | CD8: +1 | CD8: 0 | CD8: +1 | CD8: 0 |  |  |
| #16 | - | - | 0 | 0 | - | - | - | CD4: +1 | CD4: 0 | CD4: +1 | CD4: +1 | 0 | **3** |
|  |  |  |  |  |  |  |  | CD8: 0 | CD8: 0 | CD8: 0 | CD8: 0 |  |  |
| #17 | 0 | 0 | 0 | 0 | 0 | CD4: 0 | CD4: +1 | CD4: 0 | CD4: 0 | CD4: 0 | CD4: 0 | 0 | **1** |
|  |  |  |  |  |  | CD8: 0 | CD8: 0 | CD8: 0 | CD8: 0 | CD8: 0 | CD8: 0 |  |  |
| #18 | - | - | +2 | 0 | - | - | - | CD4: 0 | CD4: 0 | CD4: 0 | CD4: 0 | +2 | **4** |
|  |  |  |  |  |  |  |  | CD8: 0 | CD8: 0 | CD8: 0 | CD8: 0 |  |  |
| #19 | - | - | 0 | 0 | +2 | - | - | CD4: 0 | CD4: 0 | CD4: 0 | CD4: 0 | +2 | **7** |
|  |  |  |  |  |  |  |  | CD8: 0 | CD8: +1 | CD8: +1 | CD8: +1 |  |  |

|  | Tumor cells | Tumor cells | Tumor cells | Bregs | PB | Plasma | | | | | | | |
| --- | --- | --- | --- | --- | --- | --- | --- | --- | --- | --- | --- | --- | --- |
| **# of TNBC patients** | **CD24** | **CD44** | **mPD-L1** | **mPD-L1** | **CD33 prop.** | **sPD-L1** | **sPD-1** | **sPD-L2** | **sCD25** | **sCD27** | **sTIM-3** | **Gal-9** | **Neg. Value** |
| #1 | -2 | -2 | 0 | 0 | -2 | 0 | 0 | -2 | 0 | -2 | 0 | 0 | **-10** |
| #2 | -2 | 0 | 0 | 0 | -2 | 0 | 0 | 0 | 0 | -2 | 0 | 0 | **-6** |
| #3 | 0 | 0 | 0 | 0 | 0 | 0 | 0 | -2 | -2 | -2 | 0 | 0 | **-6** |
| #4 | -2 | -2 | -2 | -2 | -2 | - | - | - | - | - | - | - | **-6** |
| #5 | - | - | - | -2 | 0 | -2 | 0 | -2 | -2 | -2 | 0 | 0 | **-10** |
| #6 | 0 | -2 | 0 | 0 | -2 | 0 | 0 | -2 | -2 | -2 | 0 | 0 | **-10** |
| #7 | - | - | - | 0 | -2 | 0 | -2 | 0 | 0 | -2 | 0 | 0 | **-6** |
| #8 | 0 | 0 | 0 | -2 | 0 | 0 | 0 | -2 | 0 | 0 | 0 | 0 | **-4** |
| #9 | - | - | - | 0 | -2 | 0 | 0 | 0 | -2 | 0 | -2 | -2 | **-8** |
| #10 | -2 | 0 | -2 | 0 | -2 | 0 | -2 | -2 | 0 | 0 | -2 | -2 | **-14** |
| #11 | -2 | -2 | -2 | 0 | 0 | -2 | 0 | 0 | 0 | 0 | 0 | -2 | **-10** |
| #12 | 0 | -2 | -2 | -2 | -2 | 0 | 0 | 0 | 0 | 0 | -2 | 0 | **-10** |
| #13 | - | - | - | - | 0 | 0 | 0 | -2 | 0 | 0 | 0 | -2 | **-4** |
| #14 | - | - | - | - | 0 | 0 | 0 | -2 | -2 | 0 | -2 | -2 | **-8** |
| #15 | - | - | - | -2 | -2 | -2 | -2 | 0 | -2 | 0 | -2 | -2 | **-14** |
| #16 | - | - | - | - | 0 | 0 | 0 | 0 | 0 | 0 | 0 | 0 | **0** |
| #17 | 0 | 0 | 0 | 0 | -2 | 0 | 0 | -2 | -2 | 0 | -2 | - | **-8** |
| #18 | - | - | - | - | -2 | -2 | -2 | 0 | -2 | 0 | -2 | - | **-10** |
| #19 | - | - | - | 0 | 0 | -2 | -2 | -2 | -2 | 0 | -2 | - | **-10** |

Supplementary Table 2 Scoring of 18 triple negative breast cancer (TNBC) analyzed in this study. +1/+2 = positive points for beneficial factor scoring; -0.5/-2 = negative points for unfavorable factor scoring; 0 = value beneath threshold; - = not detectable.
