# Supplementary material for: Breast cancer scoring based on a multiplexed profiling of soluble and cell-associated (immune) markers facilitates the prediction of pembrolizumab therapy
Source: Cancer Cell Int. 2025 Mar 27;25:120. doi: 10.1186/s12935-025-03729-7 (PMC11948714; doi:10.1186/s12935-025-03729-7)
Supplement: Supplementary file 2 — Supplementary Material 2 [file 12935_2025_3729_MOESM2_ESM.pptx]

## Slide 1
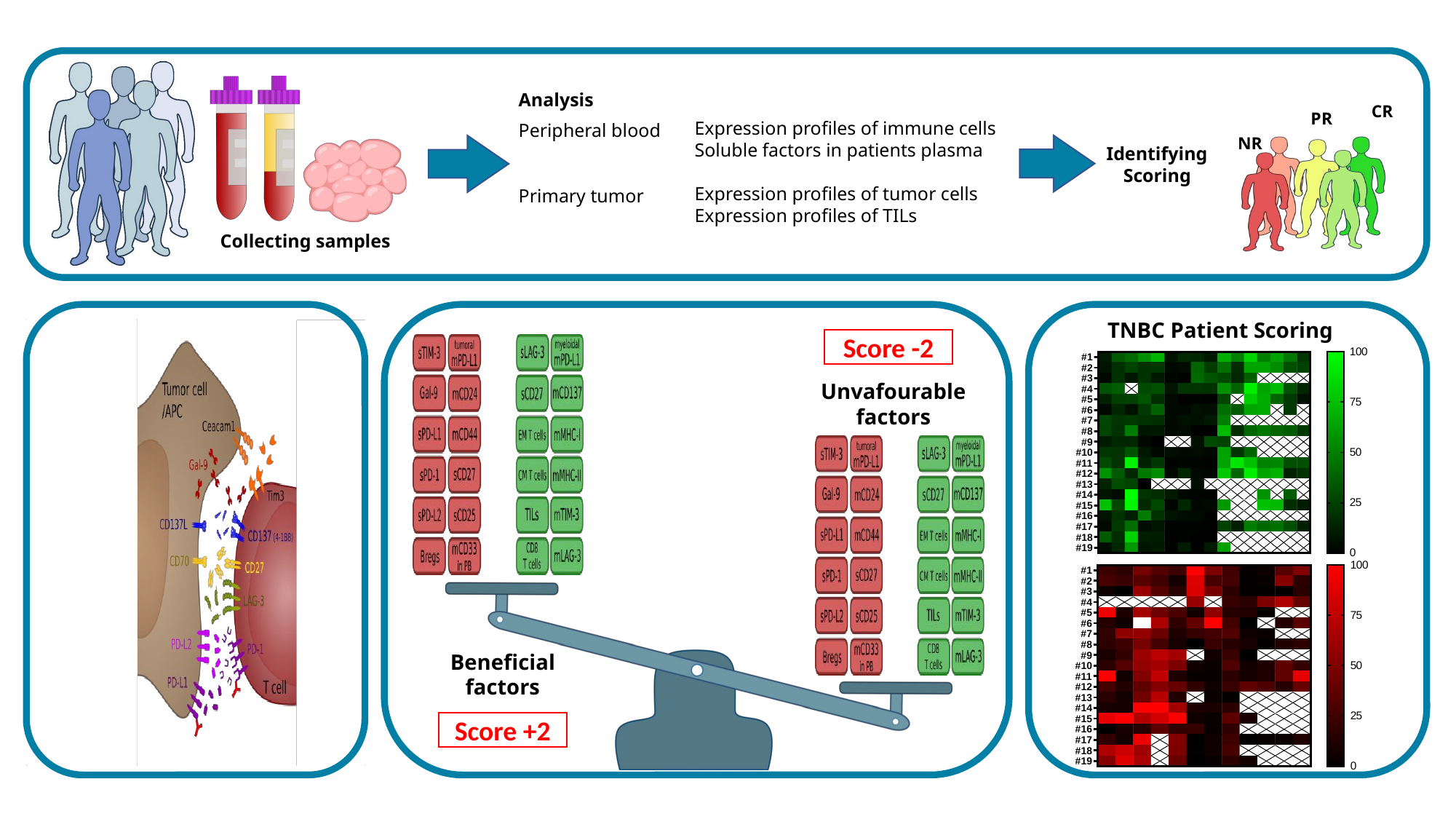

Analysis
Peripheral blood
Primary tumor
CR
PR
Expression profiles of immune cells
Soluble factors in patients plasma
Expression profiles of tumor cells
Expression profiles of TILs
NR
Identifying
Scoring
Collecting samples
TNBC Patient Scoring
Score -2
Unvafourable factors
Beneficial factors
Score +2
